# Misuse of Psychologically Active Substances of Convicts being in Prisons and their Treatment

**DOI:** 10.3889/oamjms.2016.001

**Published:** 2015-12-19

**Authors:** Safuadan Plojovic, Slavica Dimitrijevic, Andrijana Maksimovic, Sabina Zejnelagic, Adem Hurem, Muamer Muraspahic

**Affiliations:** 1 *State University of Novi Pazar, Vuka Karadzica bb, Novi Pazar, Serbia*; 2 *University of Kragujevac, Teachers College of Uzice, Trg Svetoga Save, Uzice, Serbia*; 3 *KJKP Sarajevo, Sarajevo, Bosnia and Herzegovina*

**Keywords:** misuse, psychologically active substances, convicts, prisons, treatment, addiction disease

## Abstract

Due to the data of the Ministry of Justice of the Republic of Serbia, over 70% of persons being in prisons and serving their sentences are drug addicts, and 50% of them are drug users. In the European prisons, the percentage of persons using drugs in the entire prison population is 20-70%, and in the USA 70-80%, in the Australian prisons between 50 and 80% of the convicts in prisons are addicts of psychologically active substances. The results of our survey are pursuant to official statistics data for Serbia, the European countries, USA and Australia, since 80% of our convicts in prisons have misused psychologically active substances during the period of 30 days, the previous period before coming to these institutions. More than a half of our examinees (60%), misuses narcotics and alcohol occasionally or permanently, the alcohol users only 12.7%, and only narcotics 7.3% of the ones, meaning that a treatment of addiction disease should have a significant role in prevention of recidivism.

## Introduction

Dissemination of addict disease in the world, and us, has reached such rations that we can call it an epidemic, reasonably. Statistical data of different organisations emphasise epidemiological features of drug addition, being alarming ones. The World Health Organisation conducted a research on exposure of psychologically active substances and alcohol between 2001 and 2006 (NIDA, 2007 [1]). The exposure means that a person has tried, used or still uses psychologically active substances. The research included 54,069 examinees in 17 countries, from all continents. This research had shown that a degree of usage of some substances is different. Alcohol usage over exceeds 90% in eight of seventeen countries included in. Alcohol drinking is more presented in America, Europe, Japan and New Zealand than in Africa, China, and Middle East, and the highest usage is in Ukraine (97%). The rates of drug usage is higher in the USA (cannabis 41.9%, and cocaine 16.2%), in New Zealand (cannabis 41.9%, and cocaine 4.3%) than in other countries. The rates of tobacco usage are mostly similar, being between 45-74%, except in Nigeria (16.8%) and South Africa (31.9%). There is reduction of tobacco usage in some countries.

Pursuant to the data of the *European Monitoning Centre for Drugs and Drug Addiction* (2004) [2], the percentage of drug addicts in the European prisons is from 22% to 86%. In the USA, in the period from 1984 until 1999, it was recorded an increase of drug addicts in prisons, being 3% annually (Scalia, 1999) [3]. The report from 2002 stated that in the American prisons were placed 440670 convicts, having 112447 drug addicts, 48823 for possession of drug amounts, and 56574 for possession and selling of drugs (Karberg, James, 2005 [4]). The American *Bureau of Justice Statistics* (2006) [5] stated that in 1997, 70% of convicts in the state prisons and 57% in federal prisons used drug and alcohol before being arrested. In the surveys conducted in 2002, 52% of women and 44% of men satisfy criteria to be drug addicts. In testing of juvenile convicts, it was determined that 56% of boys and 40% of girls were positive to drugs during their arresting in 2000, while in 2006, 60% of boys and 50% of girls, and this means that the number of juveniles increases, the ones who had problems with the law, but were also positive to drugs. The data from other countries also reveal a significant number of drug addicts among criminal persons. In England and Wales, 63% of male prisoners being sentenced are alcohol addicts, and 43% are drug addicts (Singleton et al., 1998 [6]). In Australian prisons, between 50% and 80% of convicts being in prisons are addicts of psychologically active substances (Australian Bureau of Criminal Intelligence, 2000 [7]).

By increasing of criminal acts related to psychologically active substances, it has been increased the number of addicts in prisons. The number of registered drug addicts being arrested in 2010 was 6211 from the entire number of 11 211, and this means 55.40%. From the entire number of convicted persons 7167, who are being served their prison sentence, 3 286 or 85% are drug addicts, while from the entire number of convicted ones 3332, 2115 or 63.48% misuse psychologically active substances (Ministry of Justice, Republic of Serbia, 2011). In the criminal structure of convicted ones in 2011, from the entire number of 7933 persons who are being served their sentence, the most frequent ones were larceny, theft, concealing and similar (1 984 convicts), drug misuse (1 688), robbery (800), body injuries (313), gun possession (244), family violence (392), raping (109), murder and murder attempts (268) and other criminal acts (1554) of convicts (Ministry of Justice, Republic of Serbia, 2012 [8]).

The situation is similar in the region. Pursuant to the Ministry of Justice of Republic of Croatia, Prison Administration Office, among 7 572 convicts who served their prison sentence in 2010, there was 22.36% of drug addicts [9]. From the entire number of convicts who were drug addicts in 2010, 55.4% made convicts who served their prison sentence in this period. During 2010, there were 1034 new persons who were received to serve their prison sentence, also drug addicts. Two years behind, in Croatia, there was an extremely high increase of criminal acts among recidivists of convicts. In 2010, even 88.59% of drug addicts who were previously sentenced, and it was 27.21% more compared to 2009, i.e. 39.63% more than in 2008 (Ministry of Justice, Republic of Croatia, Prison Administration Office, 2011 [9]).

The aim of this study was to investigate the misuse of psychologically active substances of convicts being in prisons and their treatment in the District Prison in Novi Pazar, Serbia during October and November in 2013.

## Material and Methods

The research was realised in the District Prison in Novi Pazar, Serbia during October and November in 2013. In the stated facility, male adults are being served their prison sentences, age 20 to 35. The duration of the sentences is up to12 months in prison, but usually several minor offences are joined, and thus the convicts are kept for a longer inhere. The District Prison in Novi Pazar can accept up to 80 convicts, and usually 50 of the ones serve their sentences. There is a Behavioural Service, Security Service and General Administration Service within this prison. Training Service and Health Service do not exist here, but a doctor comes on certain weekdays.

A sample makes 55 male convicts being in the prison of the District Prison in Novi Pazar at the time, speak Serbian and have agreed to participate in the research. The age of the examinees in the sample is from 20 to 60 years (AC = 29.98; CD = 8.01). The majority of the examinees live in the city (85.5%), and a smaller number in a village (14.5%). Pursuant to their nationality, the examinees can be divided into three groups: Bosniacs (76.4%), Serbs (20.0%) and Roma (3.6%). The total of 7.3% of the examinees do not have primary school finished, 54.5% have finished primary school, 12.7% has started high school, 23.6% has finished high school, and only one of the convicts has started higher education. Before their arrival to the prison, 58.2% of the examinees have been employed and 41.8% have not. The sample structure pursuant to their marriage status is: 56.4% unmarried, 7.3% are divorced or live separately, 32.7% married for the first time and 3.6% married for the second time. In Table 1, the sample structure has been presented. In Table 1, the sample structure has been presented.

**Table 1:** The Sample Structure

Variables	Categories	No.	%
Place of residence	Village	8	14.5

	City	47	85.5

Nationality	Bosniac	42	76.4

	Serb	11	20.0

	Roma	2	3.6

Schooling	Unfinished primary school	4	7.3

	Finished primary school	30	54.5

	High school started	7	12.7

	High school finished	13	23.6

	Higher education started	1	1.8

Employment	Employed	32	58.2

	Unemployed	23	41.8

Marital status	Married once	18	32.7

	Married twice	2	3.6

	Separated	1	1.8

	Divorced	3	5.5

	Not married	31	56.4

## Results

### Prevalence and the Patterns of Misuse of Psychologically Active Substances

The results show that 80% of the convicts being in the prison misuse psychologically active substances, and 20% do not. These results are pursuant to the official statistical data for Serbia. Pursuant of the data of the Sanction Performance Administration Office, the number of addicts of psychologically active substances among persons being in prisons since 2012 was 4741 drug addicts, alcoholics 1723 or 16.85% (N10226), 2011-the number of drug addicts was 4929 or 44.43%, alcoholics 1880 or 16.95% (N11094), 2010-the number of drug addicts was 6211 or 81,08%, alcoholics were 2090 or 27.28% (N7660), in 2009-the number of drug addicts was 4495 or 41.64%, alcoholics 1674 or 15.51% (N10795), in 2008-the number of drug addicts was 6063 or 62.50%, alcoholics 1695 or 17.47% (N9701), in 2007-the entire number of addicts of psychologically active substances was 73.35% (N = 6580), 53.07% (N = 4189) in 2006, 31.67% (N = 2559) in 2005 (Ministry of Justice of the Republic of Serbia, 2013 [8]).

The data on prevalence of misuse of psychologically active substances among the examinees from the sample has been presented in Table 2.

**Table 2:** Prevalence of Misuse of Psychologically Active Substances (PAS)

Misuse of PAS	No.	%
Exists	44	80.0
Does not exist	11	20.0
Total	55	100.0

We can notice that a number of convicts being serving their sentences Serbian in prisons misuse psychologically active substances. Prevalence of misuse in prison population in Serbia started to be over 50% in 2006, and their number grows constantly. In 2008, the number of drug addicts was 6063 or 62.50%, alcoholics 1695 or 17.47% to have its culmination in 2010, when it had the scope of epidemic, where from the entire number of convicts (7660), there were 6211 drug addicts or 81.8%, alcoholics 2090 or 27.28%, and its number was constant in 2011-drug addicts 4741, alcoholics 1723 and in 2012-drug addicts were 4929, alcoholics-1880. As we have already mentioned, the similar situation of increase of prevalence of misuse in prison population is similar in the region, either. In Croatia, the number of addicts of psychologically active substances being in their prisons was 349 in 2002, while in 2011-it was 3303 (Ministry of Justice of the Republic of Croatia, 2013), and this means it has increased nine times [9].

Each psychologically substance affects in a specific way, and it means it causes a specific type of addiction (Henslin, 1996 [10]). Table 3 contains data on prevalence of misuse of certain kinds of psychologically active substances. The examinees have been classified depending of whether they just use narcotics, only alcohol or a combination of narcotics and alcohol or they do not misuse psychologically active substances.

**Table 3:** Prevalence of Misuse of Certain Types of Psychologically Active Substances

Type of PAS	No.	Percentages
Narcotics	4	7.3
Alcohol	7	12.7
Narcotics and alcohol	33	60.0
Do not misuse PAS	11	20.0

Total	55	100.0

More than a half of the examinees (60%) misuse a combination of drugs and alcohol either temporarily or permanently, but only 12.7% use just alcohol and 7.3% only narcotics of all examinees. The drug addicts using any of drugs in great amounts (high-frequency users) probably use several types of drugs in combination with alcohol (Elliot et al., 1989 [11]).

Since a majority of the examinees combine different psychologically active substances, the use of certain psychologically active substances has been identified by a survey in the time period prior their arrival to serve sentences (30 days and 6 months). The data is presented in the Table 4. In the last 30 days prior their arrival to this facility, the examinees mostly used alcohol (41.8%), cannabis (38.2%), heroin (34.5%), hypnotics (29.1%) and cocaine e (29.1%). In the same period, the examinees have used other psychologically active substances: methadone (5.5%), analgetics (25.5%), barbiturates (5.5%), amphetamine (7.3%), and LSD have been used by only one examinee (1.8%). In the period of six months prior their arrival to serve their sentences, the examinees have mostly used alcohol (69.1%), heroin (63.6%), cannabis (60%), cocaine (45.5%), analgetics (32.7%) and hypnotics (30.9%). A lower frequency of usage of the following psychologically active substances has been discovered: methadone (12.7%), barbiturates (10.9%), amphetamine (14.5%), LSD (5.5%) and inhalers (5.5%). The majority of the examinees mostly used alcohol, heroin, cannabis and hypnotics in both observed periods. Numerous researches have shown that within a number of recidivists we can find over drinking or alcohol addiction (Maruna, Immarigeon, LeBel, 2004 [12]). The Sanction Performance Administration Office has warned onto a high number of alcoholics among the prison convicts. The number of registered alcoholics among persons being in prisons for serving their sentences was 697 persons in 2005, while in 2010-this number was 2090, and in 2012-the number was 1723 (Ministry of Justice of the Republic of Serbia, 2013 [8]).

**Table 4:** Distribution of the Examinees Pursuant to the Last Time Period of Usage of Psychologically Active Substances

The time of the last PAS usage	Yes		No		Total	
	No.	%	No.	%	No.	%
Getting drunk in the last 30 days	23	41.8	32	58.2	55	100
Getting drunk by alcohol in the last six months	38	69.1	17	30.9	55	100
Heroin usage in the last 30 days	19	34.5	36	65.5	55	100
Heroin usage in the last 6 months	35	63.6	20	36.4	55	100
Methadone usage in the last 30 days	3	5.5	52	94.5	55	100
Methadone usage in the last 6 months	7	12.7	48	87.3	55	100
Analgetic usage in the last 30 days	14	25.5	41	74.5	55	100
Analgetic usage in the last six months	18	32.7	37	67.3	55	100
Barbiturate usage in the last 30 days	3	5.5	52	94.5	55	100
Barbiturate usage in the last 6 months	6	10.9	49	89.1	55	100
Hypnotics usage in the last 30 days	16	29.1	39	70.9	55	100
Hypnotics usage in the last 6 months	17	30.9	38	69.1	55	100
Cocaine usage in the last 30 days	16	29.1	39	70.9	55	100
Cocaine usage in the last 6 months	25	45.5	30	54.5	55	100
Amphetamine usage in the last 30 days	4	7.3	51	92.7	55	100
Amphetamine usage in the last 6 months	8	14.5	47	85.5	55	100
Cannabis usage in the last 30 days	21	38.2	34	62.8	55	100
Cannabis usage in the last 6 months	33	60	22	40.0	55	100
LSD usage in the last 30 days	1	1.8	54	98.2	55	100
LSD usage in the last 6 months	3	5.5	52	94.5	55	100
Inhalants usage in the last 30 days	1	1.8	54	98.2	55	100
Inhalants usage in the last 6 months	3	5.5	52	94.5	55	100

Our data is also the same as the official statistics of indicators of the Ministry of Justice Republic of Serbia, on what are the most frequently used substances. Marihuana uses in average 40% and 80% (2008-65%, 2009-89%, 2010-60%, 2011-40%) (Ministry of Justice of the Republic of Serbia, 2013 [8]).

Pursuant to a report of the Treatment Service, Ministry of Justice, Prison Administration Office of the Republic of Croatia [9], the structure of the examinees for their primary psychologically active means N = 209 in 2007 was as following: the most often used psychologically active substance was heroin 82% (N = 172), cannabis 11% (N = 22), amphetamine 4% (N = 8), cocaine 3% (N = 7), 2009-heroin made 40%, cannabis-14%. For the year 2010-active substances were 44.2%, cannabis-14.3%; in 2011-active substances were 28.8% and in 2012-active substances were 39.14%, cannabis-18% (Ministry of Justice of the Republic of Croatia, 2013 [9]), and all these results point onto primary distribution of heroin as a far more often used psychologically active substance in our areas and the ones nearby.

A high frequency of heroin usage among convicts matches with the researches that heroin stimulates aggression, and in this way, criminal behaviour of addicts of psychologically active substances. With heroine users, there is a high rate of criminal, and with other drug adducts, the intensity of crminal behaviour varies with the intensity of drug usage (Anglin, Speckart, 1986 [13]). Adult criminals, multiple offenders that have used heroin before being 16 years old, also participated in different robberies and thefts (Chaiken, Chaiken, 1982 [14]). A previous beginning of heroin and cocaine usage gives a higher possibility for these addicts to become serious offenders as adults (Colins, Bailey, 1987 [15]). Regular heroin users can sustain of criminal actions and continue to use it as a part of everyday life activates, though it stimulates criminal behavior and brings energy within its users (Hanson et al., 1985 [16]). Cocaine has stimulation effects and causes skin, hearing and visual hallucinations, delirium ideas, a condition of hyper sensitivity of psycho-motor sphere and increased physical and psychological powers (Williams, 1989 [17]).

Methadone (heptanon) is misused by addicts not only as a substitution for heroin, but as a primary substance or a combination with the heroin. Methadone is also a psychologically active substance, used by the examinee addicts being in prisons for serving their sentences mostly as a treatment and quitting of heroin. Methadone substitution is being applied within addicts where there is estimation that the possibility of abstinence is very low. Some researches have shown that addicts who receive optimal doses of methadone can be more able to work, as people who do not take any medication (Munjiza, 2009 [18]). In our research, only one of the examinees has been to a methadone treatment, while the others quitted under an excuse that the one has the same addiction effects as the others. In Croatia, methadone has been used in surgeries as a substitute for heroin addiction treatment, and a medicine *subutex* has been introduced, being used more in the prison system. The main critique to methadone therapy is that the one drug is replaced by another. During 2008, 521 of convicts in prisons used this medicine (Ministry of Justice of the Republic of Croatia, 2013 [9]).

By studying patterns of drug misuse, there is knowledge on most people use illegal drugs and they are limited to a temporary usage of marihuana, while relatively small number start using other forbidden drugs such as barbiturates, amphetamine, cocaine and heroin (O’Malley, Bachman, Johnston 1988 [19]). A general characteristic of all drugs is to cause psychological addiction and have a special effect onto perception, feelings and moods (Regan, 2001 [20]).

The accomplished data on misuse of other psychologically active substances is pursuant to the official data of Ministry of Justice of the Republic of Serbia, except for cocaine. Amphetamine has been misused pursuant to the official data of Ministry-in 2008-17%, 2009-20%, 2010-40%, 2011-50%; cocaine -in 2008-5%, 2009-10%, 2010-10%, 2011-50%; ecstasy- in 2008-17%, 2009-20%, 2010-40%, 2011-50% (Ministry of Justice of the Republic of Serbia, 2013 [8]).

Pursuant to the data from Croatia, the frequency of misuse of sedatives, hypnotics and psychologically simulative drugs is 6%, and the combination with other psychologically active substances is 37.87%; 3.38% is cocaine addicts; 3.77% is stimulation means without cocaine; 11.39% is cannabis addicts; less than 0.1% uses inhalants (Prison Administration Office of the Republic of Croatia, 2007 [9]). During 2012, except all stated active substances use of 39.14% and cannabis 18%, they have used polytoxics – 30.34%, sedatives-hypnotics-7.03%, cocaine - 3.49% and stimulation means - 1.72 % (Ministry of Justice of the Republic of Croatia, 2013 [9]).

### The Treatment Need with Persons Serving Their Prison Sentences

For determination of treatment needs with convicts who do not misuse and who misuse psychologically active substances, an instrument has been used under a title *Addiction Severity Index-ASI*. It is the instrument being applied for decades in evaluation of existence and nature of this issue within criminal offenders. ASI is a relatively short, semi-structured questionnaire, designed to offer significant information on different life aspects of persons being in prisons for serving their sentences. All the gained data shall be confidential and it has been presented to all examinees in the beginning, and the data shall be only used for the scientific purposes. In the beginning of each ASI survey, there is a detailed instruction chapter for each examinee on the way of answering and to be as honest as possible, since in that way, the survey shall justify its primary purpose. The ASI test gives information on the basic aspects of life that can be connected to misuse of psychologically active substances. Life areas covered by the survey are: health status, educational and employment statues, legal status, family background, family relations and psychological status. The results of the survey shall be presented pursuant to life areas. Based on gained answers on existence, duration and intensity of the issue, it is being given a mark for each issue status: 1 – no problem, a treatment has not been indicated; 2 – smaller problems, a treatment probably has not been indicated; 3 – mild problems, a type of treatment is needed; 4 – significant problems, a treatment needed; 5 – very significant problems, a necessary treatment.

### Health Status

We have not included health issues being directly connected with misuse of psychologically active substances in evaluation in expression of certain degree of health issue status. If an examinee has a chronically disease, receives a certain therapy, and the ones does not need additional treatment, then marking is lower, since there is no need for additional treatment. A higher mark has been given if there are more frequent of long-lasting health issues, serious health problems in the last 30 days, and if the examinee wants to have a treatment. In Table 5, the number and percentage of the examinees within a group who misuse and do not misuse PAS have been presented, the ones who express a certain degree of problems in the health life area.

**Table 5:** Distribution of the Examinees who Misuse PAS and Do Not Pursuant to their Health Status

Health status	Misuse PAS		Do not misuse PAS		Total	
	No.	%	No.	%	No.	%
No problems	8	18.2	3	27.3	11	20.0
Smaller problems	14	31.8	3	27.3	17	30.9
Mild problems	12	27.3	2	18.2	14	25.5
Significant problems	9	20.5	2	18.2	11	20.0
Very significant problems	1	2.3	1	9.1	2	3.6
**Total**	44	100.0	11	100.0	55	100.0

Generally considered, it has been determined the existence of smaller problems within the majority of the examinees (30.9%), i.e. the absence of the problems (20%). This means that within half of the examinees, there is no need to apply treatment being directed towards the improvement of the health status, and within the other half, there is the need. There are a higher number of the examinees who do not have problems and those with smaller ones (54.6%), than of the examinees who misuse psychologically active substances (50%). Mild problems have been discovered within 27.3% examinees who misuse psychologically active substances and 18.2% of those who do not misuse psychologically active substances. Significant problems have existed within 20.5% of the examinees who misuse psychologically active substances and 18.2% of those who do not misuse them. Very significant health problems have had one examinee who misuses psychologically active substances and two who do not use the ones.

Pursuant to the data of the Ministry of Justice of the Republic of Serbia from 2012, the number of those being sick in prisons is the following: 4353 of the musculoskeletal diseases, 8461 of respiratory ones, 3825 of digestive disease, 6720 of cardiovascular diseases, and 3445 of a nervous system, 18 858 of mental disease, and the entire number is 45 662. The type and number of infection diseases among the persons being in prisons for serving their sentences in 2012 was: 20 of HIV, 2 of hepatitis A, 1569 of hepatitis B and hepatitis C, and 52 of TBC. The number of those being sick of hepatitis C within the persons being in prisons in Serbia grows rapidly. From 2005 until 2007, the number of the sick ones was 528 in 2005, while in 2006, it increased to almost three times 1431, and in 2007, the number of them was 1784, while it has been constant for five years period, and in 2012 it was 1569 (Ministry of Justice of the Republic of Serbia, 2013 [8]).

### Educational and Employment Status

By evaluating schooling of the examinees, whether the one has a profession, trade or a special skill, a permanent job in the last year, whether someone helps them socially, the source of their incomes and supporting of other ones, we have gained the following results on their educational and employment status. Juvenile criminals continue to perform criminal acts as adults, if they had problems at school as very young (irregular school attendance or leaving it), (Blumstein, Farrington, Moitra, 1985 [21]). In Table 6, the data has been shown on the issues in educational and business domain of examinees’ life domain of the ones who misuse and do not misuse PAS.

**Table 6:** Distribution of the Examinees who Misuse PAS and Do Not Pursuant to their Educational and Employment Status

Educational and employment status	Misuse PAS		Do not misuse PAS		Total	
	No.	%	No.	%	No.	%
No problems	0	0	0	0	0	0
Smaller problems	5	11.4	2	18.2	7	12.7
Mild problems	21	47.7	8	72.7	29	52.7
Significant problems	15	34.1	1	9.1	16	29.1
Very significant problems	3	6.8	0	0	3	5.5
Total	44	100.0	11	100.0	55	100.0

The highest is the number of the examinees with mild problems (52.7%) and significant problems (29.1%), making more than 2/3 of the total number of the examinees. It emphases a low level of educational and business status of the examinees and their need for education treatment and business education, but also a classification of the ones. Mild and significant problems (more than 80%) have had the examinees that misuse and do not misuse psychologically active substances. The number of 18.2% examinees has smaller problems and they do not misuse psychologically active substances, and 11.4% use the ones. Mild problems have 47.7% of the ones using psychologically active substances, and 72.7% of the examinees do not use the ones. Significant problems have had 34.1% of the examinees who misuse psychologically active substances, and 9.1% of the ones who do not use them. Significant problems have not had the ones who do not misuse psychologically active substances, while 6.8% had been the ones who misuse psychologically active substances with very significant problems.

### Legal Status

The evaluation of the legal status has been performed based on the current and previous verdicts of the examinees, and we have also determined the type of criminal acts and their recidivism. Per a legal model, violation of law can be considered deeds related to drug, such as usage, possession and production (Goldstein, 1985 [22]). There are criminals who committed numerous criminal acts and they do not use drug (Innes, 1988 [23]). The data describing legal status of the examinees has been shown in Table 7.

**Table 7:** Distribution of the Examinees who Misuse PAS and Do Not Misuse PAS pursuant to their Legal Status

Legal status	Misuse of PAS		Do not misuse PAS		Total	
	No.	%	No.	%	No.	%
No problems	0	0	0	0	0	0
Smaller problems	3	6.8	2	18.2	5	9.1
Mild problems	14	31.8	6	54.5	20	36.4
Significant problems	24	54.5	3	27.3	27	49.1
Very significant problems	3	6.8	0	0	3	5.4
**Total**	44	100.0	11	100.0	55	100.0

It has been determined that smaller problems with the law have 49.1%, and mild problems have 36.4% of all examinees. It tells us that within the majority of the examinees, there is need for treatment, directed to the legal remedies and different programmes being applied with the aim not to do the same criminal acts and other ones, i.e. not to have recidivism. Mild problems with the law have 54.5% of the examinees who do not misuse psychologically active substances, while the ones who do misuse these substances are 31.8% of the examinees. Significant problems with the law has 54.5% of the examinees who misuse psychologically active substances, and 27.3% has the same problems but they do not misuse them. A smaller and very significant problem has 6.8% per each group of the ones who misuse psychologically active substances, while the marks have not been given for the prisoners who do not misuse the ones.

### Family Background

In Table 8, there has been presented data on family background of the examinees, i.e. whether some of the closer siblings and family members have had problems with alcohol usage or significant psychological problems, and have been sent to treatment and hospitalisation. In case any of the examinees’ siblings do not use psychologically active substances, either has some mental disorder, it has been given a mark “no problems”. The mark “very significant problems” has been given in the cases where several siblings and family members have serious problems with the misuse of psychologically active substances or chronically mental disorders, and thus had been treated in the hospital in the past.

**Table 8:** Distribution of the Examinees who Misuse Pas and Do Not Misuse PAS pursuant to Family Background

Family background	Misuse PAS		Do not misuse PAS		Total	
	No.	%	No.	%	No.	%
No problems	16	36.4	6	54.5	22	40.0
Smaller problems	11	25.0	2	18.2	13	23.6
Mild problems	15	34.1	1	9.1	16	29.1
Significant problems	2	4.5	2	18.2	4	7.3
Very significant problems	0	0	0	0	0	0
**Total**	44	100.0	11	100.0	55	100.0

If we reconsider a family background of the examinees, we can determine that a high percentage of them do not have problems (40.0%), smaller have problems (23.6%), while mild problems has 29.1% of all examinees. In the categories with smaller and mild problems, there are a higher number of the ones who misuse psychologically active substances (59.1%), than those who do not misuse psychologically actives substances (27.3%). Significant problems have 18.2% of the examinees who do not misuse psychologically active substances, and 4.5% of those who misuse the ones. Very significant problems do not have any of the examinee categories.

Identification with social, deviant and criminal features of family members makes a base for a future behaviour of the person. A family member has a chance to learn on such behaviour from the early years of the childhood, and thus, the one has the impression of a hereditary feature. Family pathology and difficulties in its functioning show onto the background of such behaviour of an individual. Juvenile convicts continue to perform hard criminal acts if they originate from poor families, if they have someone in a closer family with a criminal behaviour, if they have a low IQ and inadequate parents’ care (Blumstein, Farrington, Moitra, 1985 [24]). The gained data in the research of the family background of the examinees are pursuant to the data in nearby countries. In a survey conducted in the territory of Bosnia and Herzegovina, in the criminal facilities in Banjaluka, Tuzla, Foca and Zenica, from a hundred of the examinees who misuse psychologically active substances, three parents of the ones also use them, while twenty-seven parents do not misuse psychologically active substances. Within 68 examinees addicted to psychologically active substances, there have been 38 parents being alcohol addicts, and 30 who has not been (Korac, 2008 [25]).

### Family and Social Relations

During the evaluation of interpersonal relations, we have tried to evaluate whether family issues would exist if there were no misuse of psychologically active substances within the families of the examinees. Based on the data presented in Table 9, the issues on the area of family relations of the examinees can be reconsidered.

**Table 9:** Distribution of Examinees who Misuse PAS and Do Not Misuse PAS regarding their Family and Social Relations

Family and social relations	Misuse PAS		Do not misuse PAS		Total	
	No.	%	No.	%	No.	%
No problems	1	2.3	0	0	1	1.8
Smaller problems	6	13.6	4	36.4	10	18.2
Mild problems	19	43.2	5	45.5	24	43.6
Significant problems	18	40.9	2	18.2	20	36.4
Very significant problems	0	0	0	0	0	0
**Total**	44	100.0	11	100.0	55	100.0

Within the highest number of the examinees, there have been mild problems (43.6%) or smaller ones (36.4%), while insignificant problems have had 18.2% of the examinees. This data speak on the need for treatment application in the aim of improvement of interpersonal relations and overcoming of the problems existing due to it. There have been a higher percentage of significant problems within the examinees who misuse psychologically active substances (40.9%) than to those who do not misuse psychologically active substances (18.2%). There has been almost the same presence with mild problems within both groups of the examinees (43.2% and 45.5%). The absence of problems in interpersonal relations has been recorded only with one examinee who misuses psychologically active substances. The gained data has been similar to the data in the surveys made in our nearby countries, and they shall be reconsidered in the parts on differences of the examinees regarding their socio-demographic and criminal-penology features.

### Mental Status

In the evaluation of psychiatrically status and need for treatment, we have reconsidered only the symptoms that have not been directly connected with misuse of psychologically active substances. In Table 10, the data on mental (psychiatrically) status has been presented.

**Table 10:** Distribution of examinees who misuse PAS and do not misuse PAS regarding their mental status

Mental status	Misuse PAS		Do not misuse PAS		Total	
	No.	%	No.	%	No.	%
No problems	4	9.1	3	27.3	7	12.7
Smaller problems	8	18.2	2	18.2	10	18.2
Mild problems	18	40.9	3	27.3	21	38.2
Significant problems	8	18.2	3	27.3	11	20.0
Very significant problems	6	13.6	0	0	6	10.9
**Total**	44	100.0	11	100.0	55	100.0

Mild problems have had 38.2%, and significant ones 20% of all examinees. Smaller problems have been discovered within 18.2% of the examinees, and lack of problems with 12.7% of the examinees. Very significant problems have had 10.9% of the total number of the examinees, and all of them misuse psychologically active substances. None of the examinees who do not misuse psychologically active substances had any significant problems of this type. Within the group of the examinees who misuse psychologically active substances, mild problems have had 40.9%, significant problems and smaller ones 18.2% per each group, 13.6% has had significant problems, and 9.1% has not had any problems. Within the examinees who do not misuse psychologically active substances, 27.3% has had mild and significant problems each, or have been without problems, and 18.2% has had smaller problems.

The gained results of the research tells that within a high number of the examinees, there have been needs for application of treatment directed towards improvement of psychological and emotional condition. The official data of the Ministry of Justice of the Republic of Serbia in all prisons for serving sentences show on the presence of similar problems. According to this source, a total number of sick ones from mental disorders in the facilities for serving prison sentences was 3893 in 2007, and 29386 of special psychiatrically check ups were performed, while in 2011, there were 27202, and in 2012-18858 (Ministry of Justice of the Republic of Serbia, 2013 [8]), and this is the highest number of check ups being performed with convicts.

### Concluding Thoughts

Misuse of psychologically active substances in the population of adult convicts in Serbian prisons has reached alarming size. Prevalence of misuse of psychologically active substances in prison population grows significantly and increases 20% per year. Pursuant to the data of Prison Administration Office among persons in prisons and addicts of psychologically active substances were 31% in 2005, 53% in 2006, and even 73% in 2007; while in 2010 of the total number of 7660 those 6211 or 81.08% were drug addicts, 2009 were alcoholics or 27.28%. It is a similar situation in the entire region. In Croatia, the number of convicts being addicted to psychologically active substances was 31% in 2005, 41% in 2006, being 41% of the prison population, and this percentage is approximately constant in this level, in 2010-it was 44.2%; and in 2012-it was 39.14%. In the European prisons, the percentage of convicts who misuse psychologically active substances is up to 80%, and similar data is valid for the USA and Australia. The relation of a high frequency misuse and high frequency of criminal actions is intensive and a long-term (Nurko, 1988 [26]). Most people who use drugs change during time and have a higher participation in criminal activities (Newcomb, Bentler, 1988 [27]).

The research results emphasise onto a necessity of introduction of wide programmes of prevalence and treatment of addiction disease, primary since a high number of addicts serving their prison sentences, but also for the other convicts who are surrounded by misuse and trade of psychologically active substances. Having the Strategy for Struggle against Drugs in Prisons, the country of Serbia and its management structures in prison system have chosen clearly for taking measures and programmes for prevention of trade and misuse of psychologically active substances in prison-treatment facilities. Unfortunately, numerous progressive ideas being in this document have not been realised until present days. It is obvious that Serbian punishment system has stayed closed to many innovations and positive experiences from the countries that marked the last decades of 20th century.

The very character of these institutions for performance of criminal sanctions is being negatively reflected onto persons who are in prisons (Plojović, S. 2013 [28]). The sense of being jeopardised is one of the most often feelings within prisoners (Johnson, 2002 [29]). An alternative position, developed mainly by criminologists, is that imprisonment is not simply a “cost” but also a social experience that deepens illegal involvement (Cullen et al., 2011 [30]). The improvement of practice in Serbian penalty institutions must start urgently to be applied, in the domain prison facilities allow it, with consultations with relevant scientific institutions dealing with this issue. The priority should be given to prevention of misuse of psychologically active substances in prisons and treatment of addicts. In the contemporary expert and scientific circles, there is an opinion that prison sentence offers ideal conditions for intervention, so as to reduce availability of psychologically active substances, and convince a convict to have an appropriate treatment. Some treatment models can look as mechanisms of social pressure onto prisoners and mechanisms of social control in a broadest sense (Garland, 2001 [31]). Having addicts at a treatment who have had their sentence verdict, they could be either voluntarily or forcedly, depending on circumstances. In the line with the practice of other countries, it can be recommended to introduce varied treatment programmes: an oral treatment programme (methadone therapy), a religious treatment programmes with a different religious influence, individual and group psycho-therapy programme, and also a family treatment programme. Based on the conducted research, two general conclusions can be derived:

- The convicts who misuse psychologically active substances represent a special group of offenders, regarding their characteristics and needs for treatment, and they all differ due to their frequency and pattern of misuse of psychologically active substances compared to a normal population. From this, the necessity is of the following and it is that
- There is a need for application of special programmes or ways when this group is considered, and there are differences in treatment needs among the examinees that misuse and do not misuse psychologically active substances.

A special significance is the work on prevention of drug addiction disease in prisons. Since the connection of criminal and drug addition disease, all convicts regardless of their history on misuse of psychologically active substances, should be treated as a high risk population. The last researches emphasise that adult criminals often continue to use psychologically active substances and do criminal acts since there is a lack of efficient treatment and supervision (Anglin, Piper, Speckart, 1987 [32]). The misuse of psychologically active substances represents a significant factor of reflecting a criminal behaviour, and thus, the treatment of addiction disease has a significant role in prevalence of recidivism (Plojović, Maksimović, 2013 [33]). Prevention programmes being applied in the world are mostly of informative character, with the aim to introduce convict population with the causes, development flow and consequences of drug addition disease. Such programmes are not demanding, since the experts employed at prisons can realise them. The efficiency of treatment is mostly reflected in improving of psychological interventions (Rohsenow, 2004 [34]). A combined treatment is possible in prisons and some authors evaluate that a treatment of addiction disease in combination with methadone therapy, counseling and treatment reduces the use of psychologically active substances within convicts that can go under such treatment, different from those who did not have any treatment (Shewan, Dalgarno, 2005 [35]). The measures of reduction of illegal drugs in prisons must be stricter. All prisons represent the most profitable market for psychologically active substance transfer, and it increases violence risk and other misuse being visible in the illegal distribution. Since all of these, a special attention must be devoted to have certain measures to reduce illegal transfer of these substances in prisons. The evaluation on the percentage of hard drugs being used by convicts emphasise onto an urgent and efficient intervention, since if there is no intervention, the usage of psychologically active substances in Serbian prisons can be to an epidemic level.

In the end, it should be emphasised that introduction of contemporary treatment programmes of misuse of psychologically active substances demands high human and material resources, and unfortunately, some of the punishment institutions in Serbia, do not have them. Further on, a lot of programmes demand separation of the convicts being in them to have special premises, special equipment and materials necessary for the realisation of programme activities, and it is impossible to provide since Serbian prisons have too many convicts. If adequate conditions are not secured for the implementation of these programmes, it is difficult to expect to live, even those programmes being evaluated by the state as strategically significant for the struggle against drug usage in prisons.
